# Antikamnia

**Published:** 1892-03

**Authors:** J. C. Johnson

**Affiliations:** Atlanta, Ga. (Lecturer on Diseases of Children, Atlanta Medical College.)


### ANTIKAMNIA.

Our attention has been frequently called during the past year to the claims made by the progenitors of Antikamnia, and as a result after careful investigation we submit the following as a compendium of our examination of its pathological and physiological action.

The therapeutic properties are, Antipyretic, Antithermic, Analgesic and Anodyne. Klemerer of Germany, makes a distinction between antipyretics and antithermics. He says, "Antithermics act only on the temperature; that is, they influence its reduction, while antipyretics influence the *cause* of the high temperature."

Fever is an acute derangement of all functions, the most important of which are acceleration of the heart's beat, and disturbance of the circulation; nervous disturbance; elevation of the bodily temperature; disturbance of nutrition, including secretion.

These four groups of symptoms may have one or two relations. One condition may be the cause of the other, or they may all be simply the result of a common cause. The nervous disturbances of fever may be summed up as a paresis or convulsions, stupor, coma or delirium.

Jergenson has found that there is a regular diurnal variation of temperature in health, precisely similar to that which is known to occur in fever; thus, the 24 hours is, as far as human temperature is concerned, divided into a diurnal and nocturnal period.

Burdon Sanderson says: "The only material difference between the conditions is that in fever the normal is 3.267 deg. F. higher."

In health, there is in man a fixed mean and a normal temperature, having a regular rhythm, and this variation is beyond the control of all disturbing causes, which do not force the organism beyond the condition of health. The maintenance of the normal temperature and its rhythmis dependent upon the nervous system, which within certain limits controls both the production and dissipation of animal heat.

So far as our present knowledge goes, the chief factor in controlling heat dissipation is the vaso-motor nerves, including in man such nerves as control sweat secretions; these nerves being able by contracting the capillaries of the surface of the body and by drying the secretions of the skin to reduce the loss of heat to a minimum, and by a reverse action to increase it to a maximum. The only nerve centre proven to exist capable of influencing the heat production without affecting the general circulation, is situated in the pons varolii or above it, and whilst it may be a muscular vaso-motor centre, it is more probably an "inhibitory heat centre." Of whichever nature it may be, it must act through subordinate centres situated in the spinal cord.

In fever, vaso-motor paralysis, when produced, is followed by an immediate fall of temperature. Fever is, therefore, a state in which the depressing poison or a depressing peripheral irritation, acts upon the nervous system which regulates the production and dissipation of animal heat. Owing to its depressed state, the inhibition centre does not exert its normal influence upon the system, and consequently tissue change goes on at a rate which results in the production of more heat than normal, and an abnormal destruction and elimination of the materials of the tissue. At the same time the vaso-motor and other heat dissipating centers are so benumbed that they are not called into action by their normal stimulus—elevation of the general bodily temperature, and do not provide for throwing off the animal heat until it becomes so excessive as to call into action, by its excessive stimulation, even their depressed forces. The nerve centers, in some cases, seem to be completely inhibited. Antikamnia removes the pressure, by dilating the capillaries and the other vascular vessels, thus causing local congestion to disappear. It reduces the pulse rate, thereby slowing the heart. It controls the vaso-motor nerves, besides calming the whole nervous system, and thus has a general soothing effect. It is a valuable remedy as an antithermic; its action in this regard is well marked, sometimes reducing the temperature 2 deg. to 3 deg. F. in a few hours. It seems to have a better effect on the high evening temperature than upon the high diurnal temperature. An extreme degree of fever, with or without complications, is dangerous, and must be controlled; in addition to the direct subtraction of heat by cold applications, we must, with caution, have recourse to antipyretic remedies. A distinction must be drawn between fever and its pathogenic agent. Such an antipyretic as Antikamnia may not act on this agent, but may have an independent action, therefore, have only a transitory effect, or it may influence this agent in the same manner that quinine does the germ of malaria or influenza.

An additional advantage gained in typhoid fevers and all gastroenteric fevers by the administration of Antikamnia in moderate doses, is that the alimentary canal is rendered alkaline, and kept in an antiseptic condition, and this is a most important condition to maintain in the treatment of all fevers.

The best results are obtained with Antikamnia when exhibited in small doses, repeated at proper intervals, and the most desirable vehicle is sherry wine or diluted brandy.

The duration of the effect of Antikamnia is longer than that produced by any of the other coal tar derivaties. It also seems indisposed to produce sub-normal temperature, as some of the others do.

In the pyrexia produced by exposure to the rays of the sun, which is common in India and in our large cities during the summer solstice, Antikamnia, in addition to cold douches, is the best remedy. Antikamnia reduces temperature by increasing radiation of heat from the body, and diminishing heat production. It stimulates the glandular system, particularly the sudorific glands. In many cases its action as a diaphoretic is phenomenal.

In some cases it has marked action on the mammary glands, producing an increase in the flow of milk. Antikamnia can be given to children without any ill effects, and is a reliable remedy. In pertussia, it keeps the paroxysms in check, and makes the patient more comfortable than any remedy we have. The cyanosis induced by its administration is *nil*, unless there is a peculiar idiosyncrasy, which is found sometimes, producing manifest heart disturbance. These are to be overcome by stimuli, or intravenous injections of salt.

Antikamnia acts admirably in the after-pains of labor, in dysmenorrhœa, hemicrania, migraine, ordinary sick or nervous headache, in the pains of locomotor ataxia, the various neuralgias, epilepsy, and in the aching pains produced by *la grippe* and dengue. It exerts a decided beneficial influence in bronchial and pneumonic troubles, as well as the fever of phthisis.

It acts as an analgesic by obtunding the sensibilities of the vaso-motor and sensory nerves. It seems to tranquilize the ganglionic centers of the whole nervous system, and has but slight action on the brain. We mean by this, that it does not stupify or produce unconsciousness. It seems to have no disturbing influence on the kidneys. It has a happy effect in nearly all neurotic troubles, and is destined to occupy a permanent position in therapeutics.

Antikamnia is of the Amido-Benzole series, in combination, and is much to be preferred to any other of this class of derivatives.

---

MEMBERSHIP IN THE AMERICAN PHARMACEUTICAL ASSOCIATION is obtained only by election at the annual meeting. "Every pharmacist and druggist of good moral and professional standing, whether in business on his own account, retired from business, or employed by another, and those teachers of pharmacy, chemistry, and botany, who may be especially interested in pharmacy and materia medica," are eligible for membership. For blank application and further information, address Dr. H. M. Whelpley, 2729 Washington avenue, St. Louis, Mo., Chairman of the Committee on Membership.

---

TASTELESS COD LIVER OIL.—I have used Wampole's Tasteless Preparation of Cod Liver Oil during the past twelve months with much satisfaction, and consider it one of our best and most reliable tonics. It is well borne by the stomach, and can be taken continuously without the unpleasant effects usually following the use of this preparation.

J. C. JOHNSON, M. D., Atlanta, Ga.
(Lecturer on Diseases of Children, Atlanta Medical College.)

